# Use of knowledge-based restraints in *phenix.refine* to improve macromolecular refinement at low resolution

**DOI:** 10.1107/S0907444911047834

**Published:** 2012-03-16

**Authors:** Jeffrey J. Headd, Nathaniel Echols, Pavel V. Afonine, Ralf W. Grosse-Kunstleve, Vincent B. Chen, Nigel W. Moriarty, David C. Richardson, Jane S. Richardson, Paul D. Adams

**Affiliations:** a Lawrence Berkeley National Laboratory, Berkeley, CA 94720, USA; b Department of Biochemistry, Duke University Medical Center, Durham, NC 27710, USA; c Department of Bioengineering, University of California Berkeley, Berkeley, CA 94720, USA

**Keywords:** macromolecular crystallography, low resolution, refinement, automation

## Abstract

Traditional methods for macromolecular refinement often have limited success at low resolution (3.0–3.5 Å or worse), producing models that score poorly on crystallographic and geometric validation criteria. To improve low-resolution refinement, knowledge from macromolecular chemistry and homology was used to add three new coordinate-restraint functions to the refinement program *phenix.refine*. Firstly, a ‘reference-model’ method uses an identical or homologous higher resolution model to add restraints on torsion angles to the geometric target function. Secondly, automatic restraints for common secondary-structure elements in proteins and nucleic acids were implemented that can help to preserve the secondary-structure geometry, which is often distorted at low resolution. Lastly, we have implemented Ramachandran-based restraints on the backbone torsion angles. In this method, a ϕ,ψ term is added to the geometric target function to minimize a modified Ramachandran landscape that smoothly combines favorable peaks identified from non­redundant high-quality data with unfavorable peaks calculated using a clash-based pseudo-energy function. All three methods show improved *MolProbity* validation statistics, typically complemented by a lowered *R*_free_ and a decreased gap between *R*_work_ and *R*_free_.

## Abstract 2

Recent developments in *PHENIX* are reported that allow the use of reference-model torsion restraints, secondary-structure hydrogen-bond restraints and Ramachandran restraints for improved macromolecular refinement in *phenix.refine* at low resolution.

## 1. Introduction   

The productive refinement of atomic models at resolutions worse than 3–3.5 Å remains a major challenge in macromolecular crystallography. At lower resolution, electron density is often ambiguous, misleading or missing where atoms should be, making it difficult to correctly fit either backbone or side-chain conformations. Traditional global validation metrics such as *R*_work_ and *R*_free_ (Brünger, 1992*b* ▶) are increasingly less sensitive to local changes in the model as resolution decreases (Murshudov *et al.*, 1997 ▶; Kleywegt & Brünger, 1996 ▶), making model validation difficult. This limitation leads to local distortions of the main chain and to incorrect rotamers or rotamer outliers both in model building and refinement, where locally incorrect models are sterically trapped in false minima (Karmali *et al.*, 2009 ▶). As a result, refinement at low resolution has sometimes been limited to fitting rigid bodies (Sussman *et al.*, 1977 ▶) rather than full-atom refinement.

To overcome the decrease in the number of available experimental data at low resolution, outside information is required to better parameterize the working model. To this end, a number of approaches have already been developed. Fundamental principles of chemistry have long been used to produce geometric targets for macromolecular refinement, such as the target bond and angle values described in Engh & Huber (1991 ▶) and related extended libraries that include targets for torsion angles, planes and chiral centers (Vagin *et al.*, 2004 ▶). Tronrud *et al.* (2010 ▶) have recently shown that conformation-dependent bond and angle targets can further improve refined models. All-atom contact-based procedures such as Asn/Gln/His flip-correction in *REDUCE* (Word, Lovell, Richardson *et al.*, 1999 ▶) or rotamer correction by real-space refinement, both available in *PHENIX* (Adams *et al.*, 2010 ▶), can improve side-chain conformations substantially. Noncrystallographic symmetry (NCS) restraints may also be used to reduce the number of independently refined parameters when applicable and have been implemented in a variety of crystallographic refinement programs, including *PHENIX*, *CNS* (Brünger *et al.*, 1998 ▶; Brunger, 2007 ▶), *REFMAC* (Murshudov *et al.*, 2011 ▶), *TNT* (Tronrud *et al.*, 1987 ▶), *BUSTER* (Bricogne *et al.*, 2010 ▶) and *SHELX* (Sheldrick, 2008 ▶).

At lower resolution, however, the simple geometry potentials used in refinement targets are often insufficient to arrive at accurate full-atom models. Real-space and steric-based methods, conformation-dependent libraries (Tronrud *et al.*, 2010 ▶) and NCS are very useful if the model is close to correct, but much less so for poorly built starting models with significant errors. For such situations, which are common at low resolution, a number of methods have been developed to include information from higher resolution related structures or from homology models into the refinement target, thereby improving the data-to-parameter ratio by using external knowledge of the likely structure. These methods include DEN restraints in *CNS* (Schröder *et al.*, 2010 ▶), LSSR in *BUSTER* (Smart *et al.*, 2008 ▶) and external structure restraints in *REFMAC* (Murshudov *et al.*, 2011 ▶), all of which use elastic network distance restraints between nearby atoms derived from the reference model to inform the refinement.

To improve macromolecular refinement at low resolution, we have implemented three methods in *phenix.refine* (Afonine *et al.*, 2005 ▶) for model parameterization that introduce no additional refined parameters, better model the underlying physical properties of macromolecules where possible and introduce external information to effectively decrease the number of refined parameters.

Firstly, we introduce a ‘reference-model’ method in *phenix.refine* that uses a related model, ideally solved at higher resolution, to generate a set of torsion restraints that are added to the refinement energy target, conceptually similar to the local NCS restraints described by Sheldrick and coworkers (Usón *et al.*, 1999 ▶). The torsion restraints are parameterized using a ‘top-out’ function, which allows the restraints to function nearly identically to a simple harmonic restraint for values near the target while smoothly tapering off at higher values. In this manner, these restraints allow for differences between the working and reference models, such as hinge motions or local changes in backbone and/or side-chain rotamer conformations. Torsion restraints were chosen for their direct correspondence to the fold of the macromolecule and the strong correlation between torsion values and a wide range of validation criteria (Chen *et al.*, 2010 ▶), and to allow facile restraint calculation without structurally aligning the reference model to the target model in Cartesian space. Unlike simple distance restraints, torsion angles can be readily interpreted in the light of complex prior chemical knowledge such as rotamer and Ramachandran distributions. To this end, in order to facilitate convergence of the starting model to the reference model we include a routine for automated correction of rotamer outliers in the working model, by comparison with the reference model, prior to refinement.

For data sets where no related models are available, the known topology of secondary-structure elements may be used to generate additional restraints for refinement. Previous work includes a general heavy-atom-based hydrogen-bond potential introduced by Chapman and coworkers (Fabiola *et al.*, 2002 ▶), which demonstrated success in improved refinement at moderate resolution using main-chain hydrogen bonds as well as side-chain–side-chain and side-chain–main-chain hydrogen bonds. We have added automatic generation of distance restraints for hydrogen bonds in protein and nucleic acid secondary structures, which can help to enforce correct geometry at lower resolution. These can be defined automatically without user intervention, but a simple parameter syntax also allows custom annotation without the need to specify individual bonding atoms for facile customization. In the absence of user-defined restraint groups, automatic annotation of helices, sheets and base pairs is performed based on the initial geometry. An internal conversion generates individual atom pairs and removes outliers based on distance-cutoff criteria. For poorer starting models where automated methods often miss desirable hydrogen bonds, interactive tools such as *ResDe* (Hintze & Johnson, 2010 ▶) allow facile manual identification of hydrogen-bond pairs, outputting simple bond parameterizations for either *phenix.refine* or *REFMAC*.

Lastly, we describe two ϕ,ψ Ramachandran restraint methods that are primarily used to restrain the overall topology of accurate hand-built models at low resolution, as well as to improve models that are close to the correct answer. Ramachandran-plot restraints have been used previously by Kleywegt & Jones (1996 ▶) in *X-PLOR* (Brünger, 1992*a* ▶), as well as in *CNS* (Brünger *et al.*, 1998 ▶), both of which targeted the general-case Ramachandran plot. Our Ramachandran restraint functions expand upon earlier methods by including context-specific Ramachandran plots for proline, pre-proline and glycine in addition to the general case (Lovell *et al.*, 2003 ▶). The first restraint target is similar to the target used in *Coot* (Emsley *et al.*, 2010 ▶), but uses a smoothed energy landscape based on the Ramachandran plot with negative regions estimated using an all-atom steric-based calculation by *Autobondrot* (Word *et al.*, 2000 ▶). We have also implemented the target function described in Oldfield (2001 ▶), which uses simple ϕ,ψ-based distance restraints to direct outliers to the nearest allowed region. The implications and possible pitfalls of using Ramachandran-based restraints are addressed in §[Sec sec5]5.

## 2. Reference-model torsion restraints   

In the ‘reference-model’ method a restraint is added to each heavy-atom-defined torsion angle in the working model, where the target value is set to the corresponding torsion angle in the reference model. These restraints serve to direct the overall topology of the model, similar to the restraints described in Kleywegt & Jones (1997 ▶); both are an alternative to the deformable elastic network approach introduced in Schröder *et al.* (2010 ▶), in which distances are restrained instead of torsion angles. In proteins, restraints are generated for χ values, for backbone torsion angles (ϕ, ψ, ω) and for the N—C—C^α^—C^β^ and C—N—C^α^—C^β^ angles to preserve proper C^β^ geometry for each residue (Lovell *et al.*, 2003 ▶) if the corresponding residue in the reference model has suitable backbone and C^β^ geometry. For RNA and DNA, restraints are generated for all proper torsions involving heavy atoms.

### 2.1. ‘Top-out’ function for torsion restraints   

**Figure 1:**
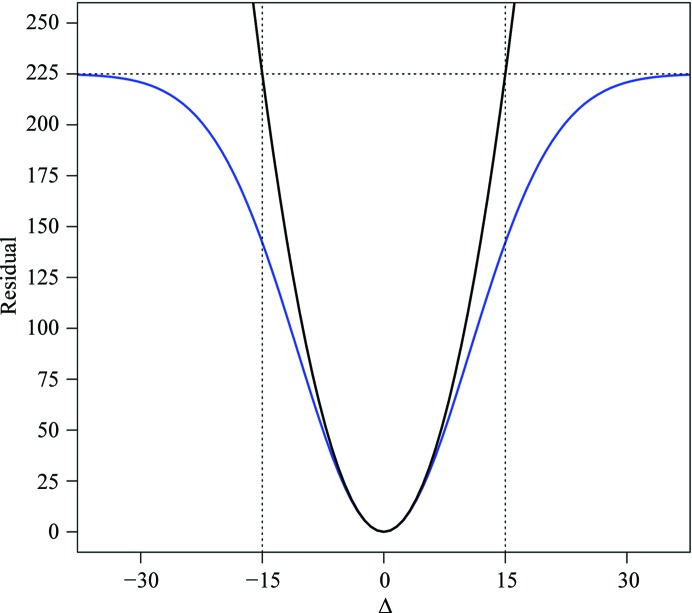
Residual plot comparing a harmonic potential with the ‘top-out’ function used for reference-model torsion restraints. For this example, *l* = 15.0° and σ = 1.0, which correspond to the default values in *phenix.refine*. The ‘top-out’ potential, shown in blue, is smoothly limited to a residual value of 225, which is equal to the value of the harmonic potential at Δ = 15.0°.

The residuals for the reference torsion restraints make use of a ‘top-out’ function, 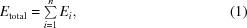


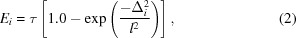


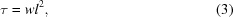


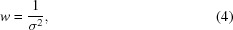
where *w* is the weight applied to each restraint, Δ*_i_* is the difference between the *i*th torsion in the working model and the corresponding torsion in the reference model, σ is a user-defined standard deviation parameter for the reference torsions and *n* is the total number of added reference restraints. For comparison, the conventional harmonic potential is defined as
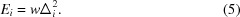
The top-out function is a variation of the Welsch robust estimator function (Dennis & Welsch, 1978 ▶) that is parameterized to be compatible with the conventional harmonic potential at values close to the minimum, similar to the Geman–McClure robust estimator function (Geman & McClure, 1987 ▶) used in *REFMAC*5 (Murshudov *et al.*, 2011 ▶) for interatomic distances. The parameter τ controls the ‘top-out’ of the harmonic potential and specifies the asymptotic threshold for the potential. The parameter *l* provides an intuitive means for the user to specify the desired top-out point, as illustrated in Fig. 1 ▶. Restraints with Δ*_i_* significantly great than *l* are effectively turned off, but remain in place in case Δ*_i_* drops below *l* during refinement. Treating the restraints in this manner allows for differences between the working model and the reference model, such as hinge motions, different domain relationships and alternate surface rotamers owing to differences in crystal packing.

The default values in *phenix.refine* are σ = 1.0° and *l* = 15. These values were determined empirically after analysis of refinements of four test series performed with a range of values for each parameter (data not shown). These test cases were PDB entries 2aea (2.58 Å)/2apj (1.6 Å), 1gtx (3.0 Å)/1ohv (2.3 Å) (see §[Sec sec2.3.1]2.3.1), 3hfl (2.65 Å)/1yqv (1.7 Å) and 4tsu (2.5 Å)/1oh0 (1.1 Å).

The *PHENIX* atom selection syntax is supported, allowing the user to specify any desired chain and/or residue-range correspondence between the working model and input reference model. In situations where there are different numbers of copies in the asymmetric unit between the two models, the user may use the same reference chain for multiple copies in the working model. Automated primary-sequence-based alignment is also supported.

### 2.2. Pre-refinement correction of rotamer outliers   

To improve the performance of refinement with a reference model, we added a complementary method to identify and correct rotamer outliers in the working model by comparison with the reference model. Outliers are identified with *phenix.rotalyze* (Adams *et al.*, 2010 ▶), which uses the Richardson rotamer distributions (Lovell *et al.*, 2000 ▶) and updates, as used in *MolProbity* (Chen *et al.*, 2010 ▶). For each outlier in the working model, if the corresponding side chain in the reference model is in a proper rotameric conformation (≥1% score), the side chain in the working model is adjusted to match the rotameric χ angles of the reference model. This pre-refinement correction step prevents badly misfitted side chains in the working model from being sterically caught in false minima, which is particularly problematic for branched or longer side chains such as Leu, Lys and Arg (Headd *et al.*, 2009 ▶). In the four test cases, the use of the pre-refinement rotamer-correction routine resulted in improvement in *R*_free_ and *R*_free_ − *R*_work_, as well as *MolProbity* statistics (data not shown). Outlier correction is the default behavior when using a reference model in *phenix.refine* and was used in all examples in this manuscript.

### 2.3. Application of reference-model torsion restraints   

#### 2.3.1. Pig 4-aminobutyrate aminotransferase   

To test the efficacy of torsion reference-model restraints, we first evaluated their impact on a pair of structures of pig 4-amino­butyrate aminotransferase, which was first solved at 3.0 Å resolution (1gtx) and later obsoleted by a 2.3 Å resolution model (1ohv), both crystallized in space group *P*2_1_ (Storici *et al.*, 2004 ▶). This sample set was chosen because the two models are of an identical protein in the same crystal form, with only the resolution of the data set differing. Furthermore, the lower resolution data set is at the higher end of our targeted range for ‘low-resolution’ data, making it an easily evaluated example for development purposes.

We refined the deposited 1gtx model against the 3.0 Å structure factors both alone and with the 1ohv model as a reference for five macrocycles with *phenix.refine*. Refinements included individual sites (atom *x*, *y*, *z*), individual ADPs, weight optimization and Cartesian NCS restraints, and did not include H atoms. The refinement that included reference-model restraints resulted in a lower* R*_free_ and *R*_free_ − *R*_work_, as well as a considerable improvement in *MolProbity* statistics. Table 1 ▶ summarizes the *MolProbity* analysis following refinement both with and without the 1ohv reference model. Substantial improvement in the clashscore percentile (Word, Lovell, LaBean *et al.*, 1999 ▶), a reduction in the percentage of rotamer outliers and an increase in Ramachandran favored are all indicative of a more realistic model, while decreases in both *R*_free_ and *R*_free_ − *R*_work_ indicate a better fit to the experimental data and less model bias, respectively.

**Figure 2:**
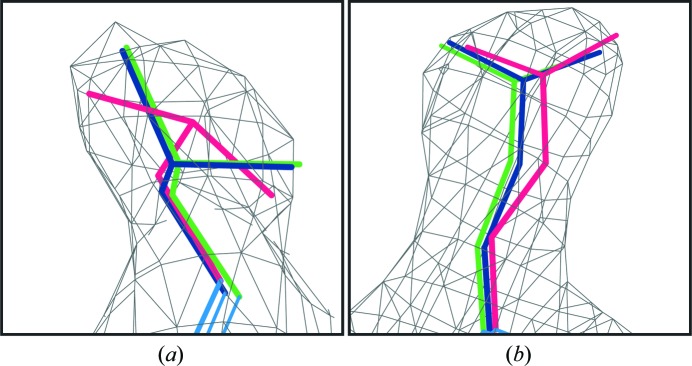
Reference-model side-chain examples. (*a*) Leu*A*34 from 1gtx/1ohv. The starting model in 1gtx (hot pink) is a rotamer outlier, while the corresponding side chain in 1ohv is a tp rotamer (green). After outlier correction and reference-model restraint generation, Leu*A*34 refines to a correct tp rotamer (dark blue). (*b*) Glu*A*41 from 1gtx/1ohv. Both the starting model in 1gtx (hot pink) and 1ohv (green) are mt-10 rotamers, but the refined position with reference-model restraints in 1gtx (dark blue) is a better fit to the density. All images were generated using *KiNG* (Chen *et al.*, 2009 ▶).

As shown in Fig. 2 ▶(*a*) and Table 2 ▶, outlier correction identifies and corrects systematic errors in the starting model by using rotamer information from the reference model. In this case, Leu*A*34 from 1gtx is originally modeled as a rotamer outlier but is corrected to a proper tp rotamer, which then refines to an energetically favorable position. Such systematic outliers are common in protein crystal structures, particularly at lower resolution (Headd *et al.*, 2009 ▶; Headd, 2009 ▶) when side-chain orientation is difficult to resolve by density fitting alone. Fig. 2 ▶(*b*) illustrates a common situation in which the Glu*A*41 side chain in the working model is in the same rotamer as the reference model, but as a result of the lower resolution data is not as ideally fitted. Restraining these side chains to the higher confidence conformation from the higher resolution reference model reduces overfitting.

#### 2.3.2. Cyclic GMP-dependent kinases   

One of the key motivations behind reference-model torsion restraints is the scenario in which two related structures, such as a protein bound to two different ligands, have both been crystallized but one crystal diffracts to a higher resolution than the other. In order to test the use of reference-model torsion restraints in this scenario, reference-model torsion restraints in *phenix.refine* were used in the refinement of a set of cyclic GMP-dependent kinases (PKGs; Kim *et al.*, 2011 ▶). Briefly, PKG Iβ was crystallized with cGMP (PDB entry 3od0), with cAMP (3ocp) and as a partial apo structure (3ogj). The cAMP-bound data set was collected to 2.49 Å resolution and a high-quality model for that resolution was determined. The cGMP (2.9 Å resolution) and partial apo (2.75 Å resolution) data sets were of lower quality and standard refinement resulted in poor models with below-average validation statistics for their respective resolutions. Owing to data-processing difficulties with the cGMP data set the usable signal only extended to 3.2 Å resolution, so refinement was carried out only to this high-resolution limit. To improve the quality of these refined models, reference-model restraints derived from the cAMP-bound model were applied to the cGMP-bound and partial apo refinements.

The results of reference-model restraint refinement in *phenix.refine* for these related structures are summarized in Table 3 ▶. Following the introduction of reference-model restraints, the models of the cGMP-bound and the partial apo forms show substantial improvement in *MolProbity* validation criteria, including boosting the clashscore percentile from 15th to 87th and from 46th to 80th for the cGMP-bound and partial apo structures, respectively, while decreasing *R*_free_ and *R*_free_ − *R*_work_ in both cases. [Note: the final models published in Kim *et al.* (2011 ▶) were refined using a development version of reference-model torsion restraints which used a truncated harmonic potential rather than the smooth top-out potential. The final models used in this study were refined using reference restraints as described in §[Sec sec2.1]2.1. As a result, the *R* values and *MolProbity* statistics presented in this study are slightly improved over the corresponding values in the PDB entry.]

#### 2.3.3. Comparison with DEN refinement   

To assess the effectiveness of our torsion-based reference-model restraints at resolutions at or below 4.0 Å and to compare their effectiveness against a related interatomic distance elastic network approach, we tested our method on 17 of the 19 models from the low-resolution data set described in Schröder *et al.* (2010 ▶). Both 1isr and 1pgf failed in reference torsion generation owing to significant bond-distance outliers in the reference-model file and were therefore excluded from this study. The DEN reference homology models were first processed with *REDUCE* to correct Asn/Gln/His flips (Word, Lovell, Richardson *et al.*, 1999 ▶). Starting models were refined both with and without reference-model restraints using *phenix.refine* (Adams *et al.*, 2010 ▶) for ten macrocycles of refinement. Refinement was carried out for individual sites in reciprocal and real space and for individual ADPs, with weight optimization for both X-ray/geometry and ADPs. All refinements were carried out using the same parameters to test the usefulness of torsion-angle restraints applied in an automated fashion and to allow fair comparison with the DEN refinements (Schröder *et al.*, 2010 ▶), which were also carried out in a singular automated fashion. It should also be noted that the DEN refinements were carried out with torsion-based simulated annealing, compared with Cartesian refinement in *phenix.refine*.

**Figure 3:**
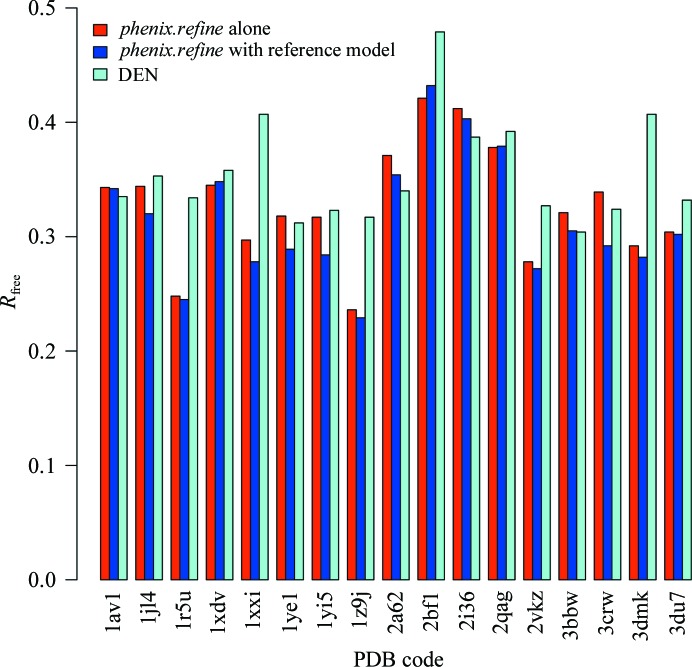
Summary of *R*_free_ improvement using reference-model restraints.

As shown in Table 4 ▶, the use of torsion reference-model restraints in *phenix.refine* produces comparable results to DEN refinement in general. Reference-model restraints result in a greater improvement in Ramachandran score for all cases, and improvements in absolute values of *R*_free_ in 13 out of 17 and in *R*_free_ − *R*_work_ in seven out of 17 cases. Seven out of 17 reference-model refined models (∼41%) improved upon the DEN results for all three metrics. Differences in *R*_free_ for *phenix.refine* alone, *phenix.refine* with reference-model restraints and DEN refinement are summarized in Fig. 3 ▶; on average reference-model restraints improved *R*_free_ by 1.2% (when compared with *phenix.refine* alone), while DEN restraints improved *R*_free_ by 1.4% [when compared with the simulated-annealing protocol described in Schröder *et al.* (2010 ▶) alone]. The consistent improvement in Ramachandran score is not unexpected. The homology models used to generate the torsion reference-model restraints all exhibit excellent Ramachandran statistics and ϕ and ψ are explicitly targeted, whereas DEN restraints do not directly optimize ϕ or ψ torsion angles.

**Figure 4:**
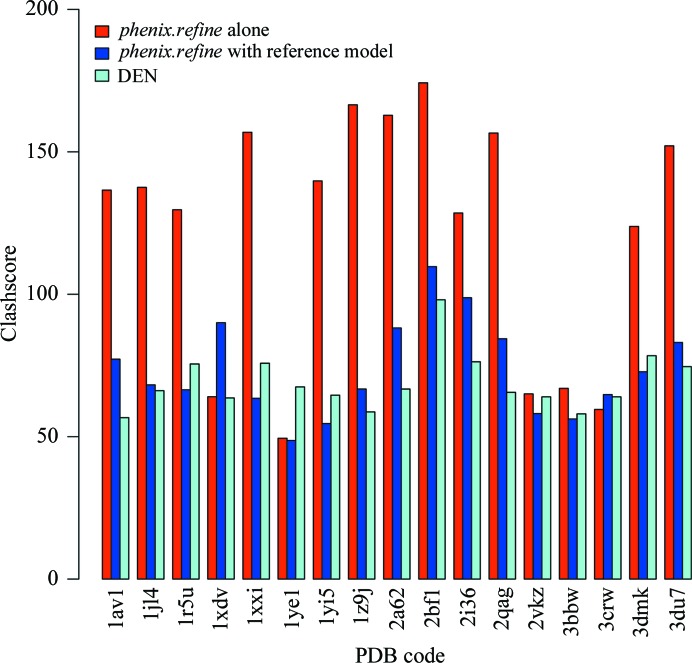
Clashscore for DEN test set following refinement in *phenix.refine* both with and without reference-model torsion restraints. As described in Chen *et al.* (2010 ▶), the clashscore is the number of clashes ≥0.4 Å per 1000 atoms.

Fig. 4 ▶ compares the clashscores for refinement with *phenix.refine* alone, *phenix.refine* with reference model restraints and DEN refinement. On average, the use of reference-model restraints reduces the clashscore by about 32%, while DEN restraints reduce the clashscore by about 35%. There were seven cases in which reference-model restraints resulted in a lower clashscore than DEN restraints (1r5u, 1xxi, 1ye1, 1yi5, 2vkz, 3bbw and 3dmk). Improved clashscore performance with DEN is not unexpected. Firstly, distance-based restraints derived from a reference model with near-ideal geometry and sterics will complement nonbonded interaction terms, reducing instances of significant steric overlap in the refined model. Further, the torsion-based dynamics used in DEN refinement may allow greater local rearrangements during refinement.

As noted in the DEN study, 1av1, 2vkz and 2bf1 all have reference models that differ from the starting model by approximately 10 Å r.m.s.d. and were included to test both the limits of the DEN method and whether or not it would have a negative impact in cases of significant difference between the reference and target models. In *phenix.refine*, the inclusion of reference-model torsion restraints decreases overfitting and improves the Ramachandran score in all three cases. *R*_free_ is slightly higher for 2bf1 compared with *phenix.refine* alone, but visual inspection of both final models reveals no major distortion following refinement with reference-model restraints. This behavior can be attributed to the relatively tight top-out potential for these restraints, which assures that only local regions of similarity are restrained in the geometry target, preventing over-biasing the working model towards the reference structure. It has been shown that DEN refinement can accommodate large domain motions between the working and reference models, *e.g.* 1xxi, 1z9j and 3crw (Schröder *et al.*, 2010 ▶), and performs well even with limited similarity between the reference and working models, *e.g.* 3dmk (only 50% similarity). Reference-model torsion restraints in *phenix.refine* perform comparably well for 1xxi, 1z9j and 3crw, but result in slightly higher overfitting for 3dmk, suggesting that in general DEN refinement may have a larger radius of convergence when there are large concerted differences between the working model and its reference.

The consistent performance of reference-model torsion restraints derived from a homology model suggests that the use of such models as references to generate torsion restraints is a productive strategy for refinement at resolutions at and below 4.0 Å.

## 3. Secondary-structure restraints   

### 3.1. Protein secondary-structure restraints   

To maintain secondary-structure elements, simple harmonic distance restraints identical in form to the covalent-bond restraints are utilized. Either the amide H or N atom may be used in conjunction with the carbonyl O atom, depending on whether or not H atoms are present in the input model. The secondary-structure elements are recorded as *PHENIX* atom selections or groups thereof rather than a comprehensive list of atom pairs, which are instead determined at runtime. Although the restraints are handled similarly to the covalent bonds and contribute to the calculation of X-ray/stereochemical weights, they are not included in the final bond statistics shown at the end of refinement and in validation.

For automatic annotation, *ksDSSP*, an open-source implementation of the Kabsch & Sander (1983 ▶) algorithm which is part of the *UCSF Chimera* package (Pettersen *et al.*, 2004 ▶), is used to generate PDB-format HELIX and SHEET records, which are converted to the format stored internally by *PHENIX*. To compensate for annotation errors, excessively long distances are filtered out of the restrained atom pairs by default using a relatively strict cutoff (see below).

### 3.2. Nucleic acid base-pair restraints   

The folds of nucleic acid macromolecules differ from those of proteins in that the main interactions that determine the tertiary structure are base-pairing interactions. The backbone of RNA in particular is considerably more flexible than the backbone of protein chains and does not provide the easily predictable hydrogen-bonding pattern associated with protein secondary-structure elements. Therefore, we parameterize hydrogen-bond restraints for nucleic acids by identifying pairs of atoms between bases within hydrogen-bonding distance and with proper geometry using *PROBE* (Word, Lovell, LaBean *et al.*, 1999 ▶). To simplify the parameter syntax, the Saenger classification (Saenger, 1984 ▶) is used to annotate bonding patterns in RNA, while DNA uses the system of Leontis & Westhof (2001 ▶). For manual annotation in cases where the starting geometry does not contain sufficient recognizable hydrogen bonds, the class may be omitted and the appropriate atoms to restrain are determined at runtime.

### 3.3. Application of secondary-structure restraints   

Tests were run using the same 19 structures from Schröder *et al.* (2010 ▶); for consistency with the DEN and reference-model refinements, explicit H atoms were not added. Three parallel refinements were performed using either the standard geometric restraints (Vagin *et al.*, 2004 ▶) alone, secondary-structure restraints with automatic annotation and default settings (N—O distance = 2.9 Å, outlier cutoff = 3.5 Å) or secondary-structure restraints with no outlier filtering. As expected, the percentage of residues forming ordered helices or sheets was better conserved in nearly every case when the additional restraints were used. An extreme example is the 1av1 structure, in which approximately 90% of residues are helical: refinement with default restraints decreased the helical content to 75%, while secondary-structure restraints with and without outlier filtering maintained this at 85 and 90%, respectively.

**Figure 5:**
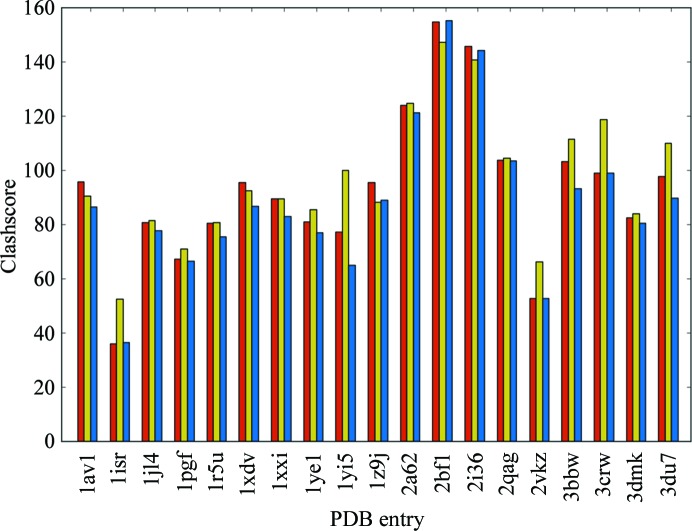
Improvement of clashscore by using secondary-structure restraints in *phenix.refine*. Red, default restraints; yellow, secondary-structure restraints without outlier filtering; blue, secondary-structure restraints with outlier filtering.

With outlier filtering, the additional restraints usually had little or no effect on *R* factors, although the gap between *R*_free_ and *R* was slightly reduced in a few structures. Ramachandran scores were marginally improved, adding on average 1.9% of residues to the favored region of the plot and eliminating 1.15% of outliers. The largest improvement was to the clashscore, which decreased by a mean of 3.8, with two structures showing decreases above 10 (Fig. 5 ▶). Eliminating outlier filtering was detrimental for nearly every structure, most likely owing to inaccurate helix assignments based on the starting model. Optimization of the hydrogen-bonding distance or increasing the outlier cutoff to 4.5 Å improved the performance for some models, but in most cases the default settings were appropriate.

## 4. Ramachandran restraints   

As an alternative to secondary-structure restraints, we also introduce Ramachandran restraints in *phenix.refine* to restrain the protein backbone. We implemented two different target functions based on the (ϕ,ψ) distributions underlying *MolProbity* (Chen *et al.*, 2010 ▶). The first is similar to the method implemented in *Coot* (Emsley *et al.*, 2010 ▶) and uses a potential function *R*(ϕ,ψ) defined by a modified Ramachandran plot with negative peaks estimated for the outlier regions using an all-atom sterics calculation (Word, Lovell, LaBean *et al.*, 1999 ▶; Chen, 2010 ▶). The second method is based on a simple harmonic restraint that strongly drives each (ϕ,ψ) outlier to the nearest point in the allowed region (Oldfield, 2001 ▶).

### 4.1. Application of Ramachandran restraints   

**Figure 6:**
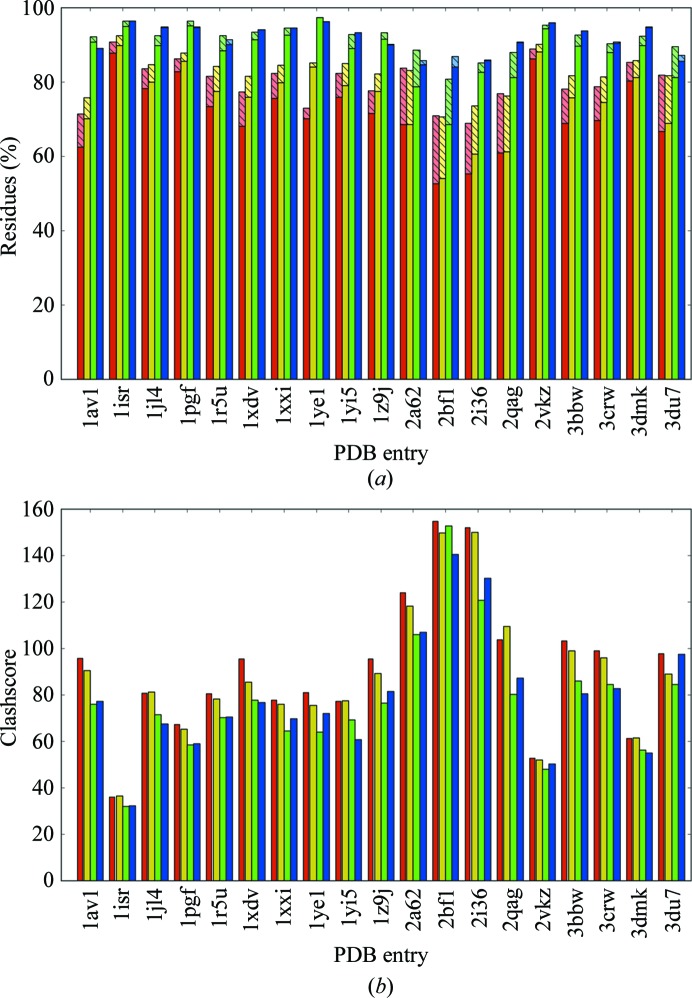
Effect of Ramachandran statistics on structure-validation criteria for 19 low-resolution structures (Schröder *et al.*, 2010 ▶). (*a*) Ramachandran percent favored and outliers. Red, default (ϕ and ψ unrestrained); yellow, monomer library uncoupled ϕ,ψ torsion restraints; green, Ramachandran restraints based on *Coot* (Emsley *et al.*, 2010 ▶) and *Autobondrot* (Word *et al.*, 2000 ▶); blue, harmonic Ramachandran restraints (Oldfield, 2001 ▶). Lower bars are percent of residues falling in the favored regions of the Ramachandran plot; upper (lighter and shaded) bars are percent outliers. (*b*) Clashscores for the same test set colored as in the first plot.

Refinements of the 19 low-resolution models in the DEN test set (Schröder *et al.*, 2010 ▶) were performed using the default *phenix.refine* strategy for five macrocycles, with the addition of automatically detected NCS restraint groups where appropriate. In addition to the default restraints and the Ramachandran potentials, a fourth set of refinements were performed incorporating torsion restraints for the protein backbone using the uncoupled ϕ,ψ values defined in the CCP4 monomer library (Vagin *et al.*, 2004 ▶). In all cases the Ramachandran statistics were significantly improved using either of the potentials, with the simple harmonic potential often eliminating all outliers, and both potentials often driving the percent favored above 90% (Fig. 6 ▶). The monomer-library separate ϕ and ψ restraints were less effective, although still an improvement on unrestrained angles, presumably owing to the omission of coupling between the two values.

The effect on *R*_free_ was less predictable and was not always correlated with the improved Ramachandran statistics. In most cases, one if not both of the Ramachandran restraint types resulted in an improved or similar *R*_free_ and reduced overfitting, but for several structures *R*_free_ increased slightly for both potentials. The clashscore was significantly and consistently improved by the use of Ramachandran restraints. Since all-atom clashes of backbone atoms are the dominant determinant of the boundary between allowed and outlier ϕ,ψ values (Lovell *et al.*, 2003 ▶), no Ramachandran criterion is independent of clashscore. Although this type of relationship degrades their independence as validation criteria, the real power of structure validation relies on the use of a large number of distinct criteria which cannot all be satisfied simultaneously by a seriously incorrect model.

## 5. Discussion   

In this manuscript, we show that the introduction of external knowledge-based information into low-resolution structure refinement generates better macromolecular models as judged by geometric and crystallographic validation criteria. The consistent success of our reference-model torsion restraints in arriving at an improved final model comparable to models of higher resolution quality demonstrate that these restraints are a viable option to improve refined models when faced with low-resolution data. Our analysis indicates that a torsion parameterization is most successful when the starting model is in the vicinity of the correct conformation, but that additional information, such as the correlated distance restraints used in the DEN method, may be needed to correct models that have very poor initial conformations far from the correct structure.

In particular, the PKG structures discussed in §[Sec sec2.3.2]2.3.2 illustrate a general scenario in which reference-model torsion restraints are invaluable, in which highly similar or identical macromolecules bound to different ligands produce X-ray data sets at varying resolutions. In this case, the 2.49 Å resolution reference model dramatically improves the final refined models of the related 3.2 and 2.75 Å resolution models, suggesting that the likely effectiveness of these restraints is in the range 3.0–3.5 Å and worse. Similar crystallographic scenarios, ranging from mutagenesis studies to pharmaceutical design and other industrial applications, will almost certainly see immediate improvement in refined models from lower resolution data sets.

At resolutions below 3.5 or 4.0 Å reference-model restraints do improve the agreement between the refined model and the structure factors and Ramachandran statistics, but the overall quality of the final model will benefit from further improvement of the methods. In particular, more comprehensive treatment of nonbonded interactions, either by all-atom contacts after hydrogen addition (Word, Lovell, LaBean *et al.*, 1999 ▶), by an empirical interaction potential such as HINT (Koparde *et al.*, 2011 ▶) or simply by a more complex hydrogen-bond potential than pure distance, may help reduce the number of clashes and otherwise improve model realism. Combining torsion-based reference-model restraints with simulated-annealing protocols or including temporary modification of geometric restraints to facilitate escape from local minima are other potential areas for improvement. More specialized extensions might include replacing the two C^α^ pseudo-torsions with a combined C^β^ deviation measure (Lovell *et al.*, 2003 ▶) and generalizing the ‘top-out’ potential to account for the periodicity of certain torsion angles and thus increasing the flexibility and the convergence radius of the reference-model restraints.

The performance of secondary-structure restraints is limited primarily by the ineffectiveness (and occasional inconsistency) of automatic annotation based on the pre-existing hydrogen-bonding geometry. As a result, elimination of excessively long bonds is essential in most cases, but this often discards legitimate regions of secondary structure that are poorly modeled. Manual annotation of the structure can potentially overcome the problem of outliers, but this is often excessively time-consuming for large structures. We are working on methods to automatically identify helices and sheets disguised by distortions, which could enable significant improvements during refinement by pulling poor initial structures into more ideal geometry. At present, however, these restraints are most useful for preserving local features of the model and introducing additional rigidity in near-final structures rather than increasing the radius of convergence early in refinement.

Use of Ramachandran restraints has two potential pitfalls. The first is a trade-off between improved statistics and the loss of an important analytical tool. Correlation of a model to the allowed regions of the Ramachandran plot has long been the standard independent validation criterion for evaluating model quality. The second, as discussed in Kleywegt & Jones (1998 ▶) and also seen in our analysis, is that restraining a poorly built model to the Ramachandran plot can sometimes move outliers into the wrong region of (ϕ,ψ) space, resulting in a model with even worse local geometry but with artificially improved validation statistics. In tests of the different potentials, we have also noticed correctly fitted side chains moved out of density as a result of overly aggressive backbone restraints. Therefore, Ramachandran restraints are most valuable when starting with a well built model where refinement at low resolution would otherwise make the model geometry worse. In cases where Ramachandran restraints are used it is imperative that this be communicated in structure deposition at the wwPDB (Berman *et al.*, 2003 ▶) and in publications.

Conceptually, the torsion reference-model method can be generalized to include other external or internal information, for example noncrystallographic symmetry restraints within a crystallographic asymmetric unit. The benefit of parameterizing NCS in this manner is that it allows automatic determination of NCS-related torsions and allows differences in related molecules at a given torsion angle or set of angles through use of the top-out potential.

This work, and the work of others (Schröder *et al.*, 2010 ▶; Smart *et al.*, 2008 ▶; Murshudov *et al.*, 2011 ▶; Kleywegt & Jones, 1997 ▶), indicates that the addition of prior knowledge into structure refinement using multiple parameterizations such as distances or torsions is capable of greatly improving the models generated at low resolution. These methods are increasingly more applicable as the database of known structures expands (Berman *et al.*, 2003 ▶). A highly interesting extension of this approach will be the incorporation of *ab initio* structure-modeling methods, which have been demonstrated to be very powerful in creating physically realistic atomic models in the absence of direct experimental data (Bradley *et al.*, 2005 ▶).

## Figures and Tables

**Table 1:** Summary of *MolProbity* validation and crystallographic statistics for refinement of 1gtx with and without 1ohv as a reference model Refinements were carried out using *phenix.refine*.

	Validation criteria	1gtx in PDB	1gtx after *phenix.refine*	1ohv	1gtx after *phenix.refine* with reference
All-atom contacts	Clashscore, all atoms	24.00	16.81	7.98	9.97
	Clashscore percentile	89th	96th	97th	97th
Protein geometry	Poor rotamers (%)	17.69	10.63	2.30	4.25
	Ramachandran outliers (%)	0.87	0.43	0.22	0.22
	Ramachandran favored (%)	95.22	95.98	97.06	96.36
	C deviation > 0.25	0	0	0	0
	*MolProbity *score	3.15	2.78	1.87	2.24
	*MolProbity* score percentile	65th	87th	94th	98th
	Residues with bad bonds (%)	0.22	0.00	0.00	0.00
	Residues with bad angles (%)	0.65	0.22	0.00	0.43
Residuals	*R*_work_	0.1869	0.1705		0.1698
	*R*_free_	0.2165	0.2123		0.2078

**Table 2:** Summary of reference-model restraint example residues Leu*A*34 is corrected *via* outlier correction to a correct tp rotamer. Glu*A*41 is restrained to the higher resolution orientation, which contributes to an overall better model.

	1gtx alone	1ohv	1gtx with reference
Leu*A*34			
_1_ ()	203.5	186.4	185.6
_2_ ()	225.6	45.6	46.3
Rotamer	Outlier	tp	tp
Glu*A*41			
_1_ ()	295.4	287.7	287.7
_2_ ()	177.1	172.6	173.0
_3_ ()	47.5	73.2	73.0
Rotamer	mt-10	mt-10	mt-10

**Table 3:** Summary of reference-model restraint refinement for related cyclic GMP-dependent kinases originally described in Kim *et al.* (2011 ▶)

	Validation criteria	cAMP-bound (2.49)	cGMP-bound (3.2)	cGMP-bound with reference (3.2)	Apo (2.75)	Apo with reference (2.75)
All-atom contacts	Clashscore, all atoms	16.53	56.57	25.79	28.52	22.51
	Clashscore percentile	81st	15th	87th	46th	80th
Protein geometry	Poor rotamers (%)	2.61	18.58	4.00	10.53	3.89
	Ramachandran outliers (%)	0.00	2.02	0.40	3.19	0.60
	Ramachandran favored (%)	98.80	85.48	96.40	89.02	96.61
	C deviation > 0.25	0	23	0	3	0
	*MolProbity* score	2.04	3.84	2.60	3.29	2.51
	*MolProbity* score percentile	95th	12th	96th	12th	86th
	Residues with bad bonds (%)	0.00	2.38	0.00	0.79	0.00
	Residues with bad angles (%)	0.00	5.95	1.18	0.98	0.39
Residuals	*R*_work_	0.1960	0.2102	0.1985	0.2205	0.2167
	*R*_free_	0.2264	0.2582	0.2389	0.2612	0.2543

**Table 4:** Comparison between reference-model restraints and DEN refinement Numbers in bold highlight values where the reference-model refinements are an improvement over DEN refinement. Bold PDB codes are models where reference-model restraints improved *R*_free_, *R*_free_ *R*_work_ and Ramachandran score over the comparable DEN refinement. Individual bold values for *R*_free_, *R*_free_ *R*_work_ and Ramachandran score are values that are an improvement over the equivalent value in DEN refinement.

		*R*_free_			*R*_free_ *R*_work_			Ramachandran score		
PDB entry	Resolution ()	*PHENIX*	*PHENIX* with reference	DEN†	*PHENIX*	*PHENIX* with reference	DEN†	*PHENIX*	*PHENIX* with reference	DEN†
1av1 ‡	4.00	0.343	0.342	0.335	0.11	0.08	0.07	0.462	**0.898**	0.840
**1jl4**	4.30	0.344	**0.320**	0.353	0.11	**0.08**	0.12	0.655	**0.803**	0.718
**1r5u**	4.50	0.248	**0.245**	0.334	0.05	**0.04**	0.05	0.627	**0.896**	0.714
**1xdv**	4.10	0.345	**0.348**	0.358	0.11	**0.11**	0.12	0.758	**0.833**	0.780
1xxi	4.10	0.297	**0.278**	0.407	0.09	0.06	0.05	0.538	**0.958**	0.842
1ye1	4.50	0.318	**0.289**	0.312	0.16	0.12	0.08	0.818	**0.975**	0.894
**1yi5**	4.20	0.317	**0.284**	0.323	0.10	**0.06**	0.07	0.608	**0.944**	0.758
1z9j	4.50	0.236	**0.229**	0.317	0.08	0.07	0.07	0.593	**0.952**	0.838
2a62	4.50	0.371	0.354	0.340	0.14	0.11	0.07	0.629	**0.749**	0.590
**2bf1** ‡	4.00	0.421	**0.432**	0.479	0.07	**0.05**	0.12	0.480	**0.666**	0.467
2i36	4.10	0.412	0.403	0.387	0.09	0.06	0.02	0.568	**0.889**	0.839
2qag	4.00	0.378	**0.379**	0.392	0.04	0.04	0.02	0.471	**0.781**	0.616
2vkz ‡	4.00	0.278	**0.272**	0.327	0.08	0.07	0.05	0.770	**0.969**	0.832
3bbw	4.00	0.321	0.305	0.304	0.08	0.05	0.01	0.806	**0.942**	0.876
**3crw**	4.00	0.339	**0.292**	0.324	0.10	**0.06**	0.09	0.781	**0.872**	0.836
3dmk	4.19	0.292	**0.282**	0.407	0.12	0.09	0.08	0.707	**0.827**	0.742
**3du7**	4.10	0.304	**0.302**	0.332	0.10	**0.07**	0.09	0.553	**0.838**	0.730
Average	4.18	0.327	0.315	0.355	0.096	0.072	0.069	0.637	0.870	0.760

† Data taken from Table 2 in Schrder *et al.* (2010 ▶).

‡ 1av1, 2bf1 and 2vkz were included in the DEN test set as controls where the reference homology model has an 10 r.m.s.d. difference from the starting model.
